# Disrupted Cross‐Scale Network Associated With Cognitive‐Emotional Disorders in Sudden Sensorineural Hearing Loss

**DOI:** 10.1111/cns.70234

**Published:** 2025-01-27

**Authors:** Biao Li, Xiao‐Min Xu, Yuan‐Qing Wu, Yuan Feng, Yu‐Chen Chen, Richard Salvi, Jin‐Jing Xu, Jian‐Wei Qi

**Affiliations:** ^1^ Department of Otolaryngology Nanjing First Hospital, Nanjing Medical University Nanjing China; ^2^ Department of Radiology Nanjing First Hospital, Nanjing Medical University Nanjing China; ^3^ Center for Hearing and Deafness University at Buffalo, the State University of New York Buffalo New York USA; ^4^ Department of Otolaryngology Nanjing Pukou People's Hospital Nanjing China

**Keywords:** cognitive‐emotional disorders, dynamic causal modeling, multilayer network analysis, structural covariance networks, sudden sensorineural hearing loss

## Abstract

**Background:**

Sudden sensorineural hearing loss (SSNHL) is associated with abnormal changes in the brain's central nervous system. Previous studies on the brain networks of SSNHL have primarily focused on functional connectivity within the brain. However, in addition to functional connectivity, structural connectivity also plays a crucial role in brain networks. Moreover, traditional functional connectivity analyses often overlook the spatial and temporal characteristics of connectivity changes and fail to provide directional information and causal relationships.

**Aims:**

This study utilized Structural Covariance Network (SCN), multilayer network analysis, and Dynamic Causal Modeling (DCM) to investigate the cross‐scale changes in neural network structure and function in SSNHL patients with accompanying cognitive and emotional disorders.

**Materials & Methods:**

We collected 3D‐T1 structural magnetic resonance image data and functional magnetic resonance image data from 70 SSNHL patients and 81 healthy controls (HCs). SCN analysis was performed based on gray matter volume, and multilayer network analysis was used to calculate node switching rates. Based on the results of multilayer network analysis, six nodes exhibiting significant inter‐group differences in node switching rates were selected as regions of interest (ROIs). DCM was then conducted to explore the causal relationships of functional connectivity between these nodes.

**Results:**

Based on SCN, there were no significant inter‐group differences in global network properties between SSNHL and HCs. At the node level, the left precentral gyrus in SSNHL showed a significant decrease in node efficiency. In the multilayer network analysis, SSNHL showed a significantly increased node switching rate at the level of the Left Superior Frontal Gyrus (L.SFG), Left Supplementary Motor Area (L.SMA), Left Superior Parietal Gyrus (L.SPG), Right Superior Parietal Gyrus (R.SPG), Right Inferior Parietal Lobe(R.IPL), and Left Thalamus (L.THA). Furthermore, the node switching rate of L.SFG showed a significant negative correlation with the Self‐Rating Anxiety Scale (SAS) scores. DCM analysis of these six nodes revealed differences in the functional effective connectivity between the left superior parietal gyrus (L.SPG) and the left supplementary motor area (L.SMA), which were positively correlated with the AVLT‐delay scores.

**Discussion:**

These findings suggest that SSNHL patients experience structural and functional remodeling of the cerebral cortex, with hearing loss leading to the reallocation of cognitive resources.

**Conclusion:**

This provides new insights into understanding the potential mechanisms between cross‐scale networks and cognitive‐emotional disorders in SSNHL.

## Graphical abstract

We propose an analysis of cross‐scale networks. Structural covariance network, dynamic causal modeling, and multilayer networks have been conducted. Our study indicates the potential mechanisms between cross‐scale networks and cognitive‐emotional disorders in sudden sensorineural hearing loss.

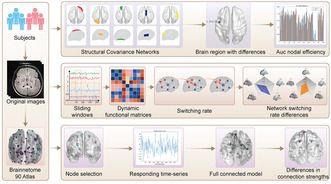

## 1 Introduction

Sudden sensorineural hearing loss (SSNHL) which is frequently encountered as an otolaryngological emergency is characterized by an inexplicable reduction in hearing capacity (a decline of at least 20 dB at two consecutive frequencies) within a 72‐h timeframe [1]. Most patients present with unilateral sudden hearing impairment, whereas bilateral occurrences are relatively rare. Associated symptoms often include tinnitus, vertigo, and a sensation of ear fullness, which considerably diminish the patient's quality of life. The incidence of SSNHL is estimated to be between 6 and 31 cases per 100,000 individuals [2]. Notably, patients with SSNHL have a 1.29‐fold increased likelihood of developing depression compared to the general population [3]. Moreover, numerous studies have established hearing loss as a contributory factor for dementia, impacting essential cognitive functions [4, 5, 6]. These findings suggest that SSNHL is more than a disease of peripheral nervous system, and it also encompasses significant alterations in the brain's central nervous system.

With the development of imaging techniques, Magnetic Resonance Imaging (MRI), as a non‐invasive neuroimaging technology, has significant advantages in sensory deprivation‐related diseases. Whether using regional homogeneity, cerebral blood flow analysis [7, 8], or brain network functional connectivity analysis [9], previous studies on cortical plasticity in SSNHL have mainly focused on brain function. While numerous studies have shown that prolonged hearing loss leads to structural changes in the cortical area [10, 11], the structural changes in the cortical region during the early stages of SSNHL remain unclear due to the acute nature of this condition and its relatively short course. Only a few studies have demonstrated that SSNHL patients with a disease course of less than one month exhibit changes in cortical gray matter volume and white matter fiber structure [9, 12, 13]. However, disappointingly, previous research has only focused on morphological changes in the cerebral cortex of hearing loss patients, without further exploring changes in brain structural connectivity. While studies have confirmed the presence of structural and functional changes in SSNHL, single‐modal connectivity analysis can no longer satisfy the complex patterns of this disease. As essential components of brain connectivity, both functional and structural connectivity are interdependent. Structural connectivity serves as the anatomical foundation of functional connectivity, while functional connectivity represents its external manifestation. Moreover, structural connectivity has high predictive value for brain connectivity [14]. Therefore, integrating structural and functional connectivity analyses provides a more comprehensive understanding of the underlying mechanisms of the SSNHL neural network.

Previous research has often assumed that the brain is a static whole, but the brain is, in fact, a complex system composed of multiple tightly connected modules [15]. Even during resting state scans, the brain's modular structure remains dynamically changing. Traditional voxel‐based morphometry (VBM) analysis primarily focuses on describing the structural features of individual voxels or brain regions, while ignoring the potential connections between brain regions [16]. Brain structural characteristics may evolve due to various factors such as natural development and aging [17]. Meanwhile, due to common neurotrophic factors and neural plasticity, structural features of certain brain regions may change synchronously with the structural changes in other regions, forming structural covariance network (SCN) [18]. SCNs are networks constructed using correlation analysis of structural features between brain regions (such as gray matter volume or cortical thickness) based on graph theory, revealing the coordinated changes between brain areas, rather than focusing solely on individual regions or structures [19]. In the brain structural network constructed by graph theory, brain regions are defined as nodes, and Pearson's correlation coefficients between structural indices of brain regions define the edges. Brain networks exhibit a “small‐world” property, which provides strong local information transmission capacity and long‐distance integration ability, ensuring efficient information transfer at both local and global levels [20]. In addition, although traditional functional connectivity analyses allow for the study of local or whole‐brain interactions, they only provide information on the strength of interactions between brain regions without revealing directional information [21, 22]. Effective connectivity addresses this limitation. Effective connectivity, based on model‐driven analysis, focuses on the interaction between brain regions, reflecting the dynamic processes of information flow and interactions between brain modules, and emphasizes causal relationships [23]. Traditional models for calculating effective connectivity include structural equation modeling, Granger causality models, and directed transfer functions. These methods assume that interactions between cortical regions are linear and analyze the output neural signals of each brain region based on this assumption [24]. However, in reality, the relationships between brain regions are nonlinear, and when the brain is exposed to external environmental stimuli or disruptions, the cortex exhibits corresponding nonlinear and dynamic functional changes. To more accurately model the dynamic functional changes of the brain, Dynamic Causal Modeling (DCM) was developed. DCM posits the brain as an “input‐state‐output” system, where external stimuli induce changes in the neural system's activity state, reflected in output signals [25]. Based on this, DCM models neuronal activity as a nonlinear dynamic differential equation system with multiple inputs and outputs [26], linking input stimuli, neuronal cluster activity states, and the recorded neural electrical activity signals. This approach constructs a biologically plausible neural network model that adheres to the interaction principles of these three components, enabling the reverse estimation of effective connectivity parameters between brain regions [27]. Initially, DCM could not be directly applied to resting‐state fMRI data until Friston introduced spectral dynamic causal modeling (spDCM), which calculates effective connectivity from resting‐state fMRI time series by observing cross‐spectra under the assumption of stationarity [28]. Subsequent studies have confirmed that spDCM in resting‐state fMRI not only provides valid estimates of model parameters but also detects group effective connectivity, neuronal fluctuations, and their amplitude differences [29]. However, it is worth noting that spDCM is a model‐driven approach, and there are limitations in ROI (region of interest) selection, as it cannot directly cover all cortical nodes in the brain for calculations. In this context, selecting appropriate ROIs becomes particularly important. Previous studies have mostly selected ROIs based on brain regions related to diseases identified by previous research or by selecting regions within default networks. This approach, however, tends to be more empirical and subjective, restricting the exploration of other brain regions relevant to the disease [30, 31]. Therefore, selecting an appropriate method for determining ROIs is crucial. Currently, research on brain functional connectivity tends to focus on brain functional networks, simplifying and highlighting the complex and heterogeneous connectivity between brain nodes. Most brain network studies utilize basic single‐layer networks, focusing on specific brain rhythms or time periods [32]. However, as mentioned earlier, brain activity is a complex, holistic process. During task‐related activities, different brain regions interact and influence each other, and single‐layer networks are insufficient to comprehensively capture the changes in brain activity, missing out on dynamic features of brain function. One of the most significant characteristics of brain functional activity is the division of labor and cooperation among multiple brain regions, and inter‐regional connectivity has been confirmed as the basis for studying brain activity. Therefore, to better investigate the distribution and dynamics of brain functional connectivity, the introduction of multilayer networks, which provide a more comprehensive representation and cover a broader range of information, is necessary [33]. The inter‐layer structure of multilayer networks can encompass the structural and dynamic features that single‐layer networks cannot express, allowing researchers to better understand the underlying clustering structure and dynamic changes and providing deeper insights into various cognitive activities in the brain. Multilayer network analysis is a novel graph‐theoretical model composed of multiple single‐layer networks. In this model, functional connectivity between nodes extends across both time and space [32]. Using a multilayer modular algorithm, neural networks are decomposed into multiple modules spanning time and space, tracking and quantifying the time variations of each node, as well as changes when switching between different modules or networks [15]. The dynamic interactions between nodes, referred to as node switching rates, can predict higher‐order cognitive processes [34]. Using this approach for selecting ROIs in spDCM not only overcomes the subjectivity and empirical nature of traditional node selection but also complements the lack of dynamic functional changes observed when selecting nodes through resting‐state functional connectivity methods [35]. With the expansion of research ideas, neuropathological studies have emphasized the role of cross‐scale networks, taking the “dynamics of resting‐state brain networks” as a key entry point. This perspective allows for the extraction of dynamic changes in the exchange of information between brain regions across time and space, providing deeper insights into the neurobiological mechanisms of diseases. Currently, there is a lack of research on the spatial and temporal dynamic changes of the cortical regions in early SSNHL patients in the resting state. The introduction of cross‐scale networks can help uncover the subtle changes in the SSNHL neural network and elucidate the temporal changes in functional connectivity and the spatial changes in structural connectivity.

In this study, we collected MRI data from both SSNHL patients and healthy controls (HCs) and aimed to unveil the dynamic alterations of SSNHL patients in both the structural and functional aspects utilizing SCN, multilayer network analysis, and DCM. Furthermore, this study also explored the correlation between these brain changes and cognitive‐emotional behaviors.

## 2 Materials and Methods

### 2.1 Subjects and Clinical Assessments

In this study, we recruited 70 patients with SSNHL and 81 HC from local community centers and otolaryngology departments of our hospitals. Both groups were well matched in terms of age, gender, and educational attainment. The informed consent was obtained for experimentation with every subject before the study.

The inclusion criteria of SSNHL were as follows: (1) 18–65 years old; (2) right‐handed; (3) at least 9 years of education; (4) a hearing threshold decline of over 20 dB at a minimum of three contiguous frequencies in both ears; (5) be within two weeks of disease onset. The exclusion criteria were as follows: (1) Ménière's disease, auditory hypersensitivity, pulsatile tinnitus, or previous ear surgeries; (2) a history of stroke or traumatic brain injury; (3) nervous system disorders or psychiatric conditions, or a family history of dementia; (4) serious cardiac, liver, or kidney diseases, chronic debilitating diseases, thyroid disorders; (5) alcohol and/or drug dependency, or current use of cognitive enhancers and psychoactive medications; (6) any contraindications to undergoing MRI scans.

Each subject underwent a series of neuropsychological assessments, including Montreal cognitive assessment (MoCA), complex figure test (CFT), auditory verbal learning test (AVLT), digit symbol substitution test (DSST), trail making test‐A (TMTA), trail making test‐B (TMTB), self‐rating anxiety scale (SAS), and Hamilton depression scale (HAMD).

### 2.2 MRI Data Acquisition

Imaging data was obtained from all participants using an eight‐channel head coil on a 3.0 Tesla Philips MRI scanner. During the scan, subjects were instructed to stay still, clear their minds, remain awake, and avoid focusing on specific thoughts. Resting‐state functional magnetic resonance imaging (rs‐fMRI) was carried out for 8 min and 8 s with gradient echo planar imaging (EPI) sequences. The parameters were: echo time (TE) = 30 ms, 36 slices of 4 mm thickness, repetition time (TR) = 2000 ms, no slice gap, field of view (FOV) = 240 mm × 240 mm, matrix size = 64 × 64, flip angle (FA) = 90°, and voxel size = 3.75 mm × 3.75 mm × 4.0 mm. Additionally, three‐dimensional turbine fast echo (3D‐TFE) T1‐weighted (T1WI) sequences were used for structural imaging, with parameters: field of view (FOV) = 256 mm × 256 mm, TR/TE = 8.1/3.7 ms, 170 slices of 1 mm thickness, no slice gap, matrix size = 256 × 256, and flip angle (FA) = 8°. The T1WI sequence acquisition time was 5 min and 29 s. All scans utilized parallel imaging with sensitivity encoding (SENSE) technology, with a SENSE factor of 2.

### 2.3 Preprocessing of MRI Data

#### 2.3.1 Preprocessing of Structural Imaging Data

Initially, the study involved converting the original DICOM format images into NIFTI format. Following this conversion, we utilized the Segment feature of the CAT12 software to segment the 3D‐MRI images [36]. This process yielded distinct images of gray matter, white matter, and cerebrospinal fluid. We then employed the DARTEL algorithm to normalize these segmented images to the Montreal Neurological Institute (MNI) standard space. This normalization facilitated the creation of detailed brain gray matter maps, which were later used for in‐depth statistical analyses and comparisons.

#### 2.3.2 Preprocessing of Functional Imaging Data

The Graph Theoretical Network Analysis (GRETNA) tool was employed in DCM analysis with the following steps [32]: Initially, the first 10 volumes of the MRI data were discarded to mitigate potential errors associated with the participants' acclimation to the scanning environment or the initial instability of the imaging equipment. Next, the inherent temporal discrepancies between slices acquired during the imaging process were adjusted using GRETNA's default slice timing correction routine. To address any issues related to head movements during scanning, the data were realigned. Specifically, any MRI data that exhibited head rotations exceeding 2° or head displacements greater than 2.0 mm were excluded from further analysis. The fourth step involved normalizing the corrected data volumes to the Montreal Neurological Institute (MNI) space. This was accomplished using an EPI template with a voxel size of 3 × 3 × 3 mm. Finally, the MRI data underwent spatial smoothing using a Gaussian kernel with a full‐width at half‐maximum (FWHM) of 6 mm, further refining the imaging data for analysis. The data obtained from the above steps were utilized for DCM analysis. For multilayer network analysis, we additionally performed the following preprocessing: First, we implemented a detrending procedure to eliminate linear trends in the data. Subsequently, a low‐frequency filtering step was applied, with the band‐pass temporal filter parameters set between 0.01 and 0.08 Hz [15]. This specific filtering range was chosen to isolate signals within this frequency band, effectively minimizing interference from other signal ranges and thereby enhancing the precision of our experimental outcomes. The final step involved the regression of various covariates, which included parameters for head motion, cerebrospinal fluid, and white matter.

### 2.4 Brain Network Connectivity Analysis

#### 2.4.1 SCN

In this study, the brain was segmented into 90 distinct regions using the Anatomical Automatic Labeling (AAL) template, and the gray matter volume (GMV) for each of these 90 regions is extracted based on this template. We perform linear regression analysis to control for the effects of sex and age. Then, we calculate the Pearson correlation coefficients of GMV between all brain regions, resulting in a 90 × 90 correlation matrix for each subject. The Brain Connectivity Toolbox (BCT) on MATLAB was employed to calculate the topological metrics of the brain's structural covariance networks for both subject groups [37]. The study's sparsity was set in the range of 0.05–0.40, advancing in increments of 0.01 [12]. The choice of minimum sparsity aimed to ensure full connectivity across all nodes in the structural networks for both groups. In terms of maximum sparsity, a value above 0.50 would push the network towards resembling a random network. We then conducted 1000 iterations of a non‐parametric permutation test to compare global and nodal differences between the two groups. Then, to confirm the non‐random characteristics of the network's topological attributes, the gray matter covariance network was contrasted against 100 generated random networks, ensuring the reliability and stability of our findings. Global network properties include clustering coefficient (measuring the degree of network cliquishness), characteristic path length (the average shortest path between nodes in the network, with a shorter Lp indicating shorter paths for information transfer), normalized clustering coefficient (Gamma) (the ratio of the real network's clustering coefficient to that of 100 random networks), normalized characteristic path length (the ratio of the real network's characteristic path length to that of 100 random networks), global efficiency (higher global efficiency indicates faster information transfer across the network, measuring the network's overall information transmission capacity), local efficiency (measuring the capacity for local information transfer), and small‐world properties (the ratio of normalized clustering coefficient to normalized characteristic path length) [37]. Nodal network properties include node betweenness (higher node betweenness indicates more information passing through that node, signifying its importance in the network), node degree (a higher node degree indicates more connections to that node), and node efficiency (evaluating the information transfer efficiency of that node, assessing each node's information transmission capacity within the network) [38].

#### 2.4.2 Multilayer Brain Network Analysis

Analysis of time‐varying multilayer networks was computed in our research. We derived the average time series for each node by averaging the rs‐fMRI signals of all voxels within each AAL90 atlas region. Using the DynamicBC software, we computed the dynamic functional connectivity of the preprocessed data, applying a sliding window method with a window length of 20 time repetitions (TRs) and a step length of 0.95 TR, creating a total of 201 time windows [15]. Pearson correlation coefficients were calculated among the 90 nodes for each time window, resulting in a dynamic network matrix (*N* × *N* × *W*) for each participant, where *N* (90) represents the number of nodes, and *W* (201) is the count of sliding time windows. We then used an iterative Louvain multilayer modularization algorithm to explore the dynamic restructuring of brain networks in each time window. This algorithm, by optimizing the modularity quality factor *Q* (ranging from 0 to 1), segmented the modules in the multilayer network. Higher *Q* values denote greater network modularization. The parameters *ω* and *γ* in this algorithm were critical for defining the extent of topological and temporal connections and for the modularity measurement [39]. A lower *ω* value indicates an increase in the network's switching rate. We executed computations with random combinations of *ω* (0.5, 0.75, 1) and *γ* (0.9, 1, 1.1) to derive the best outcomes. The node switching rate was calculated based on the percentage of time frames in which each node transitioned to different network assignments. To mitigate the randomness inherent in the iterative Louvain algorithm, we conducted 50 rounds of multilayer community detection for each subject under every combination of *ω* and *γ* parameters. The final results were determined by averaging all network statistics, including the network switching rates, across these 50 iterations.

#### 2.4.3 DCM

According to the results of multilayer network analysis, we identified six nodes to serve as regions of interest (ROIs): left dorsolateral superior frontal gyrus (L.SFG) (−22, 46, 24), left supplementary motor area (L.SMA) (−6, 12, 60), left superior parietal gyrus (L.SPG) (−22, −42, 56), right superior parietal gyrus (R.SPG) (24, −42, 56), right inferior parietal lobe (R.IPL) (48, −40, 34), left thalamus (L.THA) (−14, −18, 8) [40]. The following procedures were then implemented for these ROIs using SPM12 (available free at http://www.fil.ion.ucl.ac.uk/spm/software/spm12/) based on Matlab: (1) establish a General Linear Model (GLM) and extract the time series of all ROIs; (2) extract signals of the cerebrospinal fluid and white matter separately; (3) regressed out covariates to adjust the GLM, including the signals of cerebrospinal fluid and white matter, along with head motion parameters; (4) build a sphere model for the selected ROIs, utilizing a mask to extract the time series of the ROIs; (5) construct a fully connected model with 6 ROIs as nodes, where each pair of nodes was connected bidirectionally; (6) use cross‐spectra to do parameter estimation.

### 2.5 Statistical Analysis

#### 2.5.1 Demographic and Clinical Data Analysis

Demographic and clinical data for the two groups were analyzed using SPSS version 26.0. For data that followed a normal distribution, paired *t*‐tests were performed. For data that did not follow a normal distribution, analysis of variance (ANOVA) or non‐parametric tests were used as appropriate. A significance level of *p* < 0.05 was set.

#### 2.5.2 SCN

All graph theory parameters of SCNs were calculated using the Area Under the Curve (AUC) method to assess both global and nodal network properties, with multiple comparisons corrected using the False Discovery Rate (FDR) approach for a more comprehensive comparison of topological parameter differences between groups. For global network properties, a *p*‐value < 0.05 was considered significant. For nodal network properties, differences were deemed significant at *p* < 0.05 after FDR correction.

#### 2.5.3 Multilayer Brain Network Analysis

A two‐sample independent *t*‐test was performed using GRETNA to compare the modularity coefficient *Q* and node switching rates between the two groups, with correction for multiple comparisons using FDR. A *p*‐value < 0.05 indicated significant differences between the groups.

#### 2.5.4 DCM

We employed the SPSS 26.0 software (Chicago, IL, United States) to conduct a two‐sample *t*‐test on the node‐to‐node connection values of SSNHL compared to HC. A *p*‐value of less than 0.05 was considered indicative of significant differences between these groups.

#### 2.5.5 Relationships Between Cross‐Scale Networks and Clinical Measurements

Pearson's correlation was applied to investigate the relationships between cross‐scale networks and clinical measurements. *p* < 0.05 was considered statistically significant.

## 3 Result

### 3.1 Demographic and Clinical Characteristics

As shown in Table 1, there were no significant differences between SSNHL group and HC in terms of gender, age, and education level. The mean hearing thresholds of left ear and right ear in the SSNHL group were higher than HC (*p* < 0.001). In neuropsychological tests, SSNHL patients performed worse in AVLT‐delay and SAS scale (*p* < 0.001), indicating the SSNHL group showed cognitive impairments and anxiety.

**TABLE 1:** Demographic information and clinical characteristics.

	SSNHL	HC	*p*
Demographic information			
Number of subjects	70	81	—
Gender (male/female)	30/40	23/58	0.087
Age (years)	50.66 ± 13.93	53.49 ± 5.35	0.112
Education (years)	10.44 ± 3.11	10.64 ± 3.44	0.711
Duration (days)	5.70 ± 5.56	—	—
Neuropsychological tests			
MoCA	27.10 ± 1.68	26.73 ± 1.32	0.137
CFT	34.72 ± 1.38	34.93 ± 1.69	0.422
CFT‐delay	16.19 ± 4.28	15.12 ± 6.17	0.216
AVLT	35.37 ± 7.71	37.64 ± 8.82	0.093
AVLT‐delay	6.27 ± 1.78	7.68 ± 2.46	< 0.001
DSST	68.23 ± 9.55	68.79 ± 12.22	0.752
TMTA	58.93 ± 16.09	59.33 ± 20.21	0.891
TMTB	157.33 ± 54.11	146.75 ± 61.66	0.263
SAS	38.07 ± 8.10	33.88 ± 5.26	< 0.001
HAMD	4.39 ± 2.26	4.21 ± 2.04	0.619
Hearing thresholds of each ear			
Left ear	48.36 ± 27.35	20.91 ± 4.39	< 0.001
Right ear	50.70 ± 28.98	21.27 ± 3.78	< 0.001

*Note:* Data are expressed as mean ± standard deviation. *p* values were calculated with the independent *t* test or *x* ^2^ test, as appropriate.

Abbreviations: AVLT, auditory verbal learning test; CFT, complex figure test; DSST, digit symbol substitution test; HAMD, Hamilton depression scale; HC, healthy controls; MoCA, Montreal cognitive assessment; SAS, self‐rating anxiety scale; SSNHL, sudden sensorineural hearing loss; TMTA, trail making test‐A; TMTB, trail making test‐B.

### 3.2 SCN

#### 3.2.1 Global Network Properties

At the global network level, there were no significant differences between groups in terms of Cp, Lp, Gamma, Lambda, Sigma, Eglobal, and Elocal (*p* > 0.05) (Table 2). However, both SSNHLs and HC exhibited small‐world properties within the sparsity range of 0.05–0.40 (HC *σ* = 1.11, SSNHL *σ* = 1.40, *σ* > 1).

**TABLE 2:** Comparison of the differences in global network‐level topological indices between two groups (AUC).

	HC	SSNHL	Actual difference	*p*
Cp	0.202	0.202	−0.0001	0.987
Lp	0.843	0.799	−0.044	0.506
Gamma	0.490	0.596	0.107	0.208
Lambda	0.441	0.420	−0.021	0.292
Sigma	0.382	0.485	0.102	0.103
Eglobal	0.156	0.167	0.010	0.343
Elocal	0.243	0.245	0.002	0.777

*Note: p* values were calculated with the independent *t* test.

Abbreviations: Cp, clustering coefficient; Eglobal, global efficiency; Elocal, local efficiency; Gamma, normalized clustering coefficient; Lambda, normalized characteristic path length; Lp, characteristic path length.

#### 3.2.2 Nodal Network Properties

**FIGURE 1:**
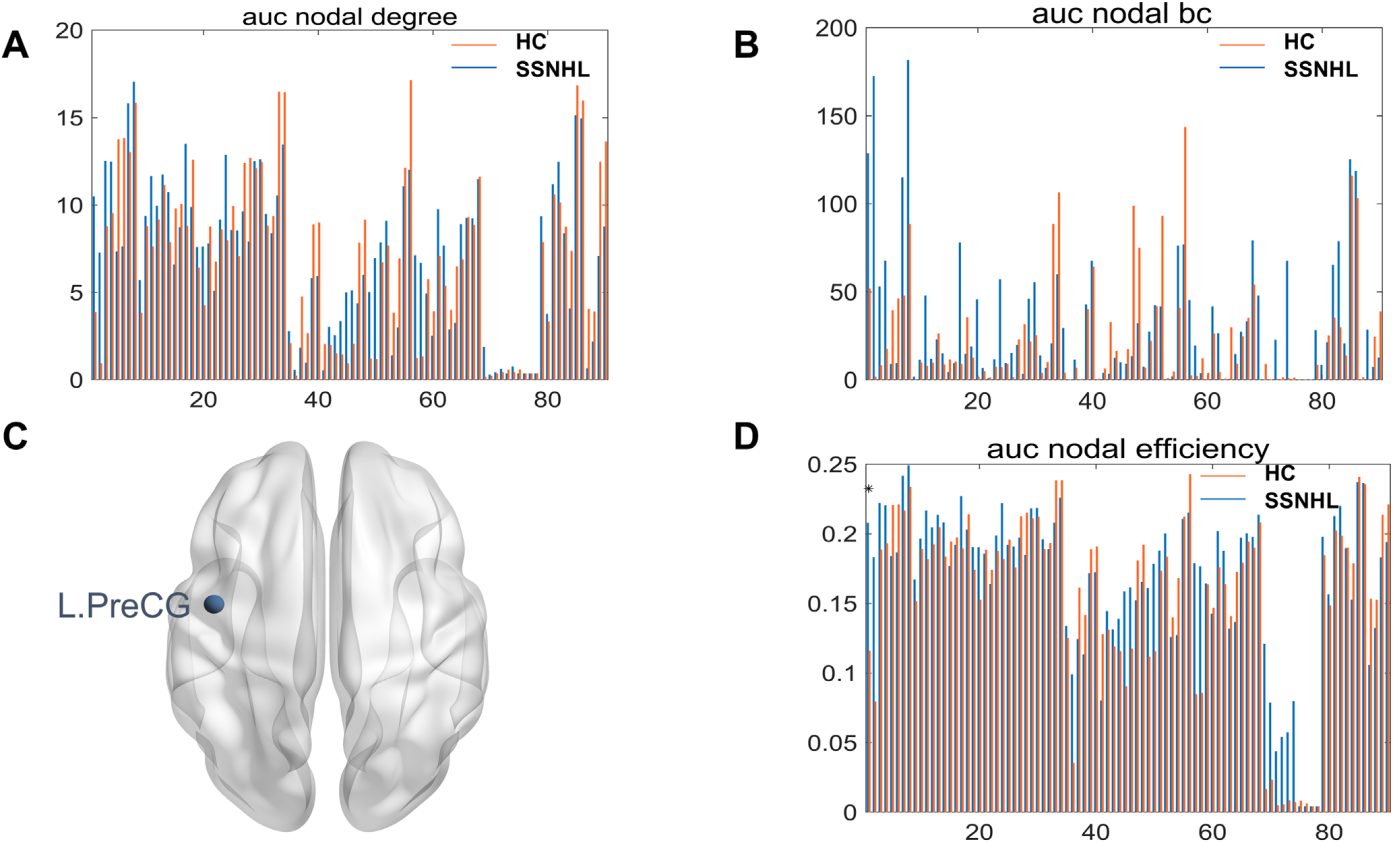
Node attribute indices of the two groups. (A) the auc node degree of two groups; (B) the auc nodal bc of two groups; (C) brain regions with differences in node efficiency between the two groups (*p* < 0.05, FDR‐corrected); (D) the auc nodal efficiency of two groups. L.PreCG, left precentral gyrus.

At the nodal level, there were no significant differences between the two groups in terms of node betweenness and node degree (*p* > 0.05, FDR corrected). However, significant differences were observed in node efficiency, specifically in the left precentral gyrus (L.PreCG) (*p* < 0.05, FDR corrected), as illustrated in Figure 1.

#### 3.2.3 Multilayer Brain Network Analysis

**FIGURE 2:**
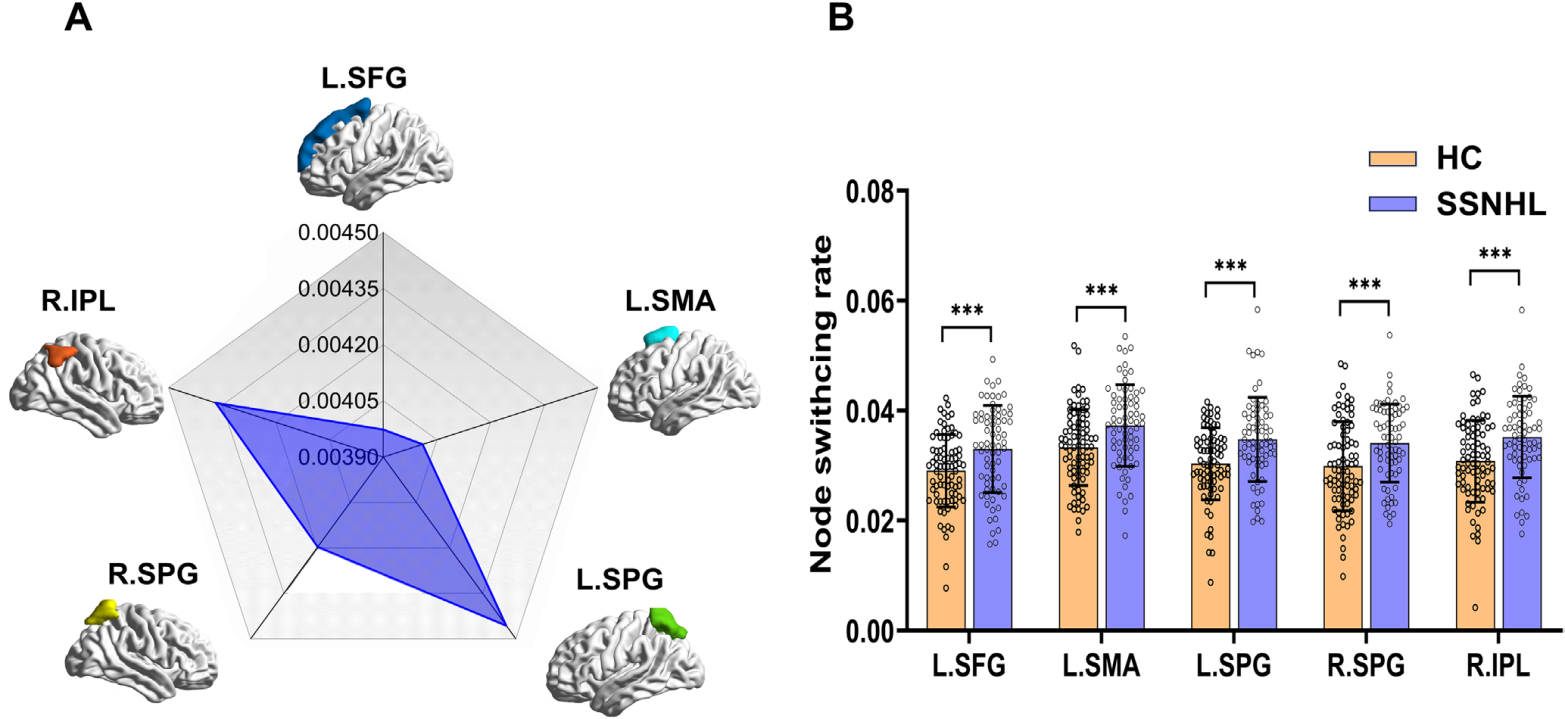
The network switching rate of two groups when gammas = 0.9, omegas = 1. (A) The network switching rate differences of the principal gradient between two groups. (B) The network switching rate of two groups (*p* < 0.001, FDR‐uncorrected). L.SFG, left superior frontal gyrus; L.SMA, left supplementary motor area; L.SPG, left superior parietal gyrus; R.IPL, right inferior parietal; R.SPG, right superior parietal gyrus.

**FIGURE 3:**
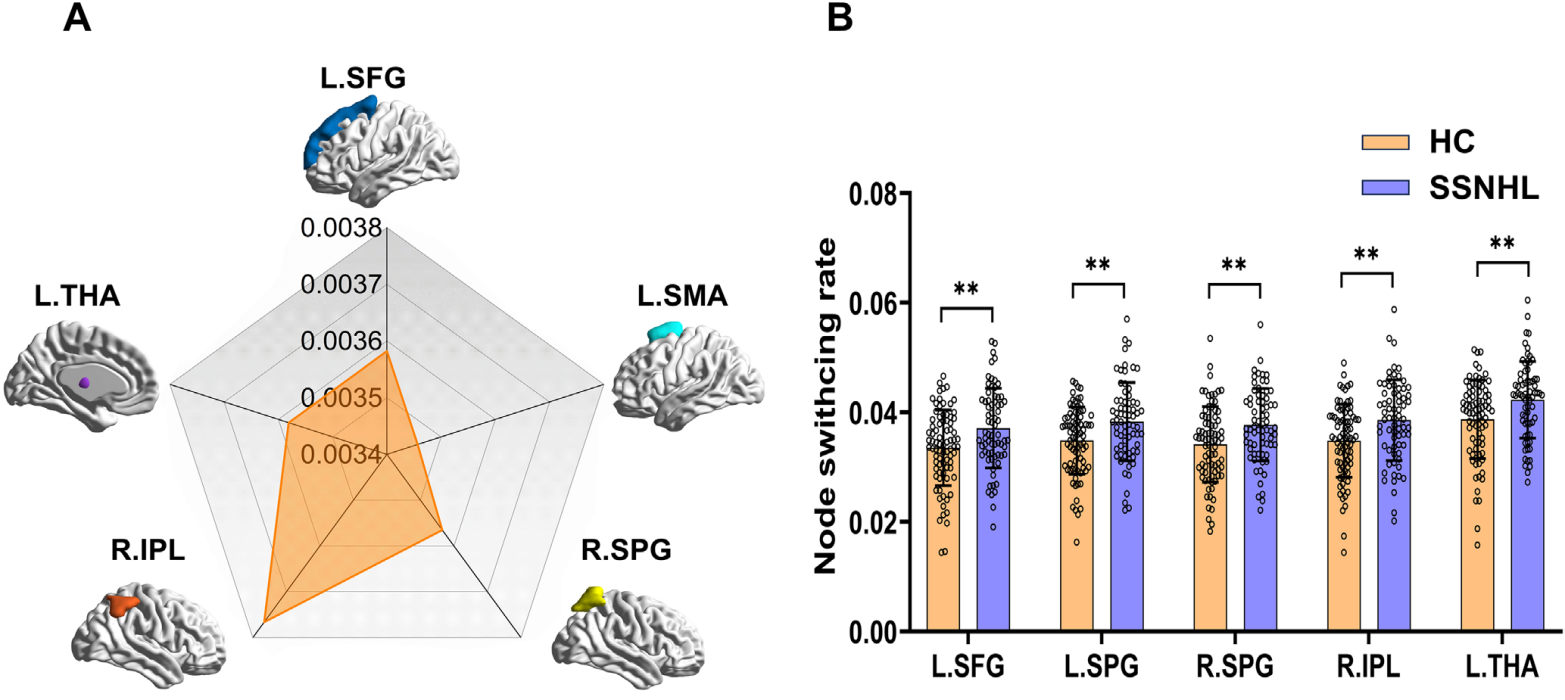
The network switching rate of two groups when gammas = 1, omegas = 1. (A) The network switching rate differences of the principal gradient between two groups. (B) The network switching rate of two groups (*p* < 0.01, FDR‐uncorrected). L.SFG, left superior frontal gyrus; L.SMA, left supplementary motor area; L.SPG, left superior parietal gyrus; R.IPL, right inferior parietal; L.THA, left thalamus.

In the setting of *ω* = 1 and *γ* = 0.9 (Figure 2), significant differences were observed in the node switching rates of L.SFG, L.SMA, L.SPG, R.IPL, and R.SPG (*p* < 0.05, FDR corrected). However, at the modular level, no significant group differences were observed (*p* > 0.05, FDR corrected). When *ω* = 1 and *γ* = 1 (Figure 3), significant differences between the two groups were noted in the node switching rates of L.SFG, L.SMA, L.SPG, R.IPL, and L.THA (*p* < 0.05, FDR corrected). Similarly, no significant differences were found at the modular level between the two groups (*p* > 0.05, FDR corrected).

#### 3.2.4 DCM

**FIGURE 4:**
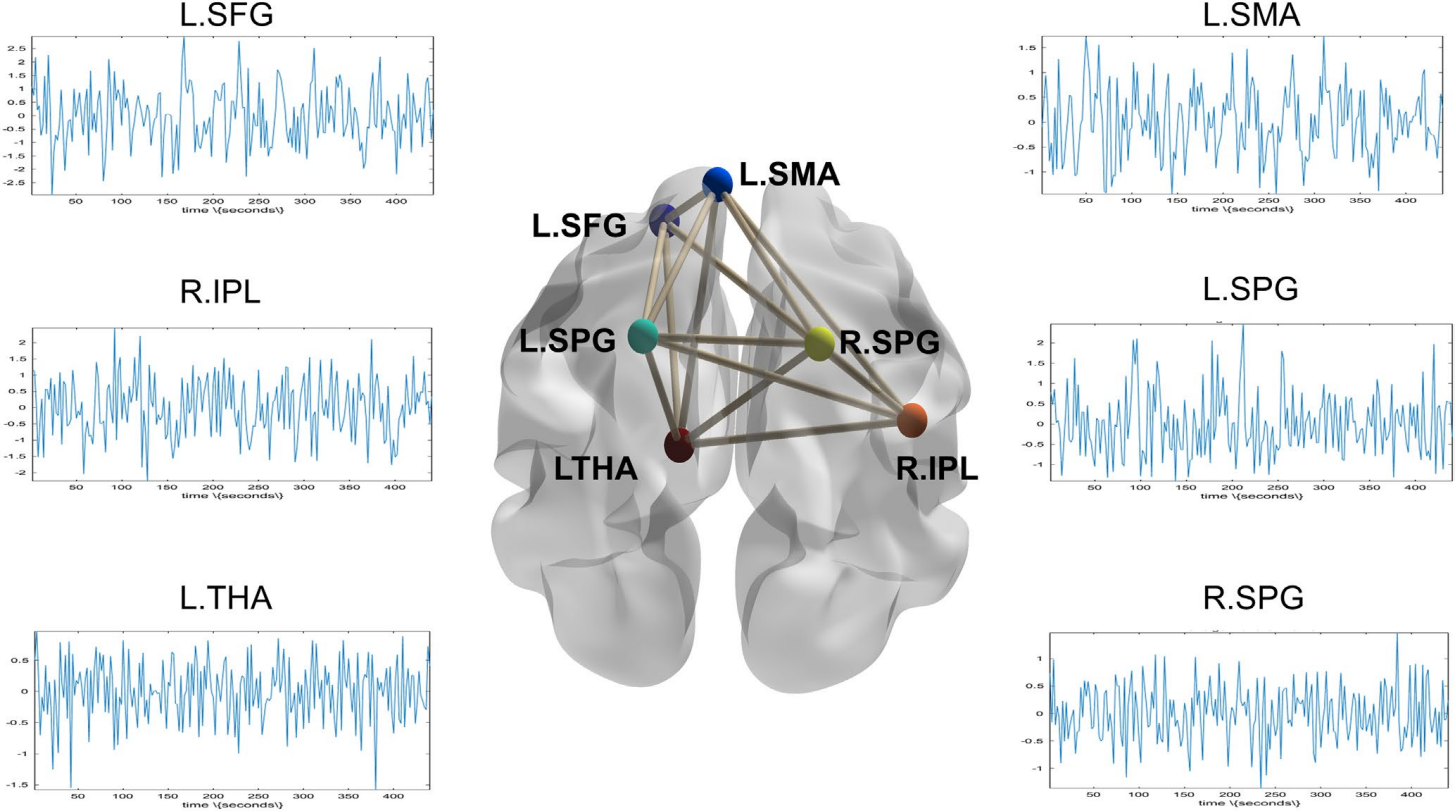
Full connected model and the responding time series of ROIs. L.SFG, left superior frontal gyrus; L.SMA, left supplementary motor area; L.SPG, left superior parietal gyrus; R.SPG, right superior parietal gyrus; R.IPL, right inferior parietal; L.THA, left thalamus.

**FIGURE 5:**
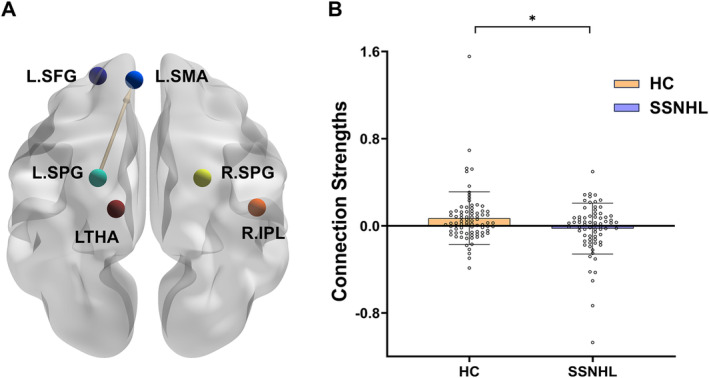
(A) Brain regions with differences in connection strengths between the two groups (*p* < 0.05); the arrow represents the direction of connection. (B) The connection strengths of two groups. L.SFG, left superior frontal gyrus; L.SMA, left supplementary motor area; L.SPG, left superior parietal gyrus; R.SPG, right superior parietal gyrus; R.IPL, right inferior parietal; L.THA, left thalamus.

Utilizing the insights from the multilayer network analysis, six nodes were identified as ROIs for DCM analysis. These nodes were integrated into a fully connected network model, depicted in Figure 4. In the fully connected model, the functional connectivity values of each node are presented in Tables 3 and 4. The two groups showed a significant difference only in the functional connectivity value from L.SPG to L.SMA (*p* < 0.05, as shown in Table 5 and Figure 5).

**TABLE 3:** Mean connection strengths (in Hz) from HC.

	L.SFG	L.SMA	L.SPG	L.THA	R.IPL	R.SPG
L.SFG	0	0.038	0.005	0.007	0.017	−0.022
L.SMA	0.202	0	0.070	−0.009	0.010	0.051
L.SPG	−0.013	−0.016	0	0.022	0.058	0.122
L.THA	0.019	−0.042	0.021	0	−0.114	0.004
R.IPL	0.096	0.002	−0.045	0.046	0	−0.054
R.SPG	0.142	0.107	0.132	−0.031	0.138	0

*Note:* In rows there are source regions, in columns—target regions.

Abbreviations: L.SFG, left superior frontal gyrus; L.SMA, left supplementary motor area; L.SPG, left superior parietal gyrus; L.THA, left thalamus; R.IPL, right inferior parietal; R.SPG, right superior parietal gyrus.

**TABLE 4:** Mean connection strengths (in Hz) from SSNHL.

	L.SFG	L.SMA	L.SPG	L.THA	R.IPL	R.SPG
L.SFG	0	0.036	0.011	0.009	0.014	−0.015
L.SMA	0.193	0	−0.026	−0.004	−0.013	0.006
L.SPG	−0.014	−0.004	0	−0.015	0.045	0.173
L.THA	0.036	−0.044	0.092	0	0.029	−0.041
R.IPL	0.134	0.079	−0.026	−0.002	0	−0.027
R.SPG	0.111	−0.016	0.105	0.031	0.086	0

*Note:* In rows there are source regions, in columns—target regions.

Abbreviations: L.SFG, left superior frontal gyrus; L.SMA, left supplementary motor area; L.SPG, left superior parietal gyrus; L.THA, left thalamus; R.IPL, right inferior parietal; R.SPG, right superior parietal gyrus.

**TABLE 5:** *p*‐value of the difference in connection strength between two groups.

	L.SFG	L.SMA	L.SPG	L.THA	R.IPL	R.SPG
L.SFG	0	0.926	0.810	0.920	0.887	0.805
L.SMA	0.848	0	0.015*	0.859	0.475	0.175
L.SPG	0.986	0.825	0	0.336	0.819	0.300
L.THA	0.882	0.983	0.464	0	0.178	0.575
R.IPL	0.623	0.083	0.675	0.196	0	0.506
R.SPG	0.718	0.114	0.745	0.185	0.438	0

*Note:* In rows there are source regions, in columns—target regions.

Abbreviations: L.SFG, left superior frontal gyrus; L.SMA, left supplementary motor area; L.SPG, left superior parietal gyrus; L.THA, left thalamus; R.IPL, right inferior parietal; R.SPG, right superior parietal gyrus.

* *p* < 0.05.

#### 3.2.5 Correlation Analysis

To further assess the relationships between cross‐scale network architectures and clinical variables, Pearson's correlation was conducted. In the SNHL group, the L.SPG‐L.SMA connectivity was positively associated with the score of AVLT‐delay (*r* = 0.335, *p* = 0.005). And the switching rate of L.SFG was negatively correlated with SAS performance (*r* = −0.404, *p* = 0.001).

## 4 Discussion

In this study, we employed methods of SCN, multilayer brain network analysis, and DCM to investigate the dynamic changes in the brain networks of patients with SSNHL from both structural and functional connectivity perspectives. At the level of structural connectivity, an increase in node efficiency was observed in L.PreCG of SSNHL patients. In terms of functional connectivity, SSNHL patients exhibited an increased node switching rate at the levels of L.SFG, L.SMA, L.SPG, R.IPL, R.SPG, and L.THA. However, a decrease in functional connectivity between L.SPG and L.SMA was noted.

SSNHL, being an acute condition, has a relatively short disease course, whereas changes in cortical structure of the brain occur over a slower process. Previous studies have found a reduction in the volume of the superior frontal gyrus only in the later stages (14–30 days) of SSNHL patients [9]. In terms of global properties, there were no abnormalities observed in structural connectivity among SSNHL patients. Additionally, both individuals with SSNHL and HC demonstrated small‐world properties. The small‐world property reflects the optimal balance between regional segregation and global integration [41], ensuring that the brain processes local and global information with maximum efficiency while consuming minimal energy [42] Surprisingly, in the present study, we discovered a significant increase in node efficiency in L.PreCG of patients with SSNHL. Node efficiency, a crucial metric for assessing structural connectivity, reflects the capability of a node to transmit information. The PrCG is uniquely involved in sensory‐motor and multi‐sensory functions related to language processing, including control of throat movements, auditory processing, and reading and writing functions [43]. Although traditionally considered part of the motor cortex and associated with motor control, evidence suggests that the PreCG also plays a role in auditory‐related events. Early functional MRI studies have shown that the PreCG is activated to varying degrees by linguistic stimuli [44]. The PreCG integrates sound with speaking actions to obtain complete linguistic information [43]. Thus, activation of the PrCG helps in speech perception under noisy or restricted auditory conditions [45]. Therefore, the enhanced node efficiency of the PreCG observed in SSNHL patients in this study may represent a compensatory response to diminished auditory capabilities. The desynchronization between sound loss and the motor cortex enhances additional sensory and motor stimulation [46]. By increasing information exchange capacity with other nodes, the brain compensates to maintain normal speech perception. Previous studies have demonstrated that patients with hearing loss show significantly higher activation of the precentral gyrus when exposed to pure‐tone stimuli [47]. Increased fluorodeoxyglucose uptake in the PrCG of SSNHL patients has been observed to meet the demands of speech discrimination tasks [48]. This suggests that the compensatory increase in node efficiency in the PrCG of SSNHL patients is reasonable. However, different findings have been reported in previous studies. For example, hearing loss patients have shown cortical thinning in the precentral gyrus [44, 49], and SSNHL patients exhibit a decrease in low‐frequency oscillation amplitude (ALFF) in the precentral gyrus [8, 50]. While these results differ, which may be related to the duration of hearing loss or patient heterogeneity, they are sufficient to demonstrate that hearing loss leads to structural and functional changes in the precentral gyrus, thereby affecting the integration of auditory and language functions. Multilayer network analysis transcends spatial and temporal limitations, effectively revealing the dynamic changes in functional connectivity within the cortical areas of the brain in patients with SSNHL [46]. Although previous studies have indicated a reduction in functional activity in the superior parietal gyrus (SPG) of SSNHL patients [8, 46] which contrasts with our findings, this also highlights the structural and functional remodeling of the SPG due to hearing loss. The SPG is a crucial brain region involved in cognitive and motor‐related processes, serving as a mediator for somatosensory processing, visuomotor integration, and spatial attention [51]. It plays a role in movement perception, particularly in obtaining speech information by observing hand or lip movements. Similarly, although the SMA is distant from the auditory system and participates in action preparation within the motor network, it also plays a role in auditory processing and auditory imagery [52]. The SMA contributes to the motor response to sound and is also involved in working memory [53]. Previous studies have demonstrated that patients with auditory hyperreactivity exhibit enhanced sensitivity of the SMA to sound stimuli [52]. In patients with age‐related hearing loss, SMA activity is enhanced during the performance of tasks with a high memory load [54]. Based on these, we speculate that the loss of hearing function results in the SMA of SSNHL patients becoming more sensitive to sound signals, allowing the body to make timely motor responses to auditory stimuli.

The IPL, as a part of the default mode network, plays a role in mediating attention and comprehending language information [55]. Additionally, the IPL is a critical area for the integration of sensory information. The THA also holds an extremely important position in the brain [56]. Past research has confirmed that hearing loss can lead to structural and functional abnormalities in the IPL [57]. The THA serves as a relay station for the majority of sensory information, such as visual, auditory, and tactile signals, to the cerebral cortex [58]. The THA is also associated with emotional and cognitive functions, particularly through its connections with the limbic system of the brain. It plays a role in memory formation and processing, especially through interactions with memory‐related structures such as the hippocampus [59]. Adverse auditory conditions lead to the additional recruitment of other brain regions, especially those involved in cognitive functions. With limited auditory information, the activation of brain regions involved in cognitive functions increases, establishing stronger connections with other nodes to assist the brain in making accurate responses. In the results of our multilayer network analysis, it is noteworthy that the node switching rate in the left superior frontal gyrus (L.SFG), an essential component of the prefrontal cortex, was significantly increased, and it showed a significant negative correlation with SAS scores. The L.SFG, as part of the prefrontal cortex, participates in the amygdala's neural circuitry, which plays a key role in the establishment and extinction of conditioned fear. The amygdala is responsible for the establishment and expression of conditioned fear, while the prefrontal cortex regulates the activity of the amygdala to suppress the intensity of fear expression [60, 61]. Damage to the prefrontal cortex or L.SFG functions or structure would likely reduce its inhibitory effect on the amygdala, leading to an anxiety state [62]. Previous studies have found a significant positive correlation between cortical thickness in the SFG and anxiety levels [63], as well as a positive correlation between fALFF values in the L.SFG and perceived stress levels [64]. This aligns with our findings, where the SFG is activated when the body perceives anxiety or stress. However, in contrast, our study found a significant negative correlation between the node switching rate in the superior frontal gyrus (SFG) and SAS scores. A structural MRI study also showed that social anxiety levels were negatively correlated with cortical thickness in the SFG [65]. This may represent a compensatory mechanism, where the body enhances its suppression of anxiety‐related structures such as the amygdala through significant activation of the SFG, in order to reduce or alleviate the anxiety response. This interpretation explains our experimental results: stronger activation of the SFG may serve to limit the intensity of the anxiety state.

Previous studies have generally selected regions of interest (ROIs) based on brain regions identified in prior research related to the disease, or by choosing certain regions from the default network. This approach tends to make ROI selection more empirical and subjective, limiting the progress of research on other brain areas associated with the disease. Some studies have used ALFF and resting‐state functional connectivity to identify disease‐specific functional nodes for effective connectivity analysis [35, 66]. Traditional resting‐state functional networks reflect the average temporal characteristics between brain regions or networks, but they fail to capture the complete dynamic changes in brain function across multiple time scales. During dynamic brain activity, as time progresses, the brain cortex transitions from one stable state to another, representing the switching between multilayer brain networks. In each layer of the brain network, nodes are connected to their counterparts in other layers, revealing connections between adjacent time points. Therefore, in this study, we first used multilayer network analysis to identify specific nodes of functional changes in the SSNHL brain cortex and used these nodes as ROIs for DCM analysis to obtain optimal positive results. As expected, the results showed that SSNHL exhibited reduced effective functional connectivity between the SPG and SMA. Research by Tomiyama et al. [67] found that the functional connectivity between the SMA and the parietal lobe is associated with impaired motor response inhibition in patients with Obsessive‐Compulsive Disorder. This suggests that the connection between the SMA and the parietal lobe may play a role in motor control and inhibition. The connection between the SPG and the SMA is also strengthened during tasks involving imagined musical performance. These studies indicate that functional connectivity between the SPG and the SMA may play a significant role in language processing, cognitive functions, and the integration of multimodal information [68]. Patients with SSNHL, due to their limited ability to receive auditory information, show cognitive anomalies as auditory signals fail to establish a connection with motor instructions. Furthermore, in the subsequent correlation analysis, the effective connectivity between the SPG and SMA was positively correlated with the AVLT‐delay scores, further supporting our conclusion. In the spDCM analysis of this study, the effective connectivity model for SSNHL showed a reduction in effective connectivity from the SPG to the SMA, while no significant changes in effective connectivity were observed between other nodes. There are several possible reasons for this: First, the multilayer network analysis results showed that the six identified nodes exhibited more active switching rates. This compensatory response helped mitigate the effects of hearing loss on the body. This compensation was reflected not only in the increased node switching rates but also in the maintenance of normal levels of effective connectivity between nodes. Second, it may also be related to our method of ROI selection. While multilayer network analysis can provide dynamic activity information of nodes over time, it primarily reflects the state switching of individual nodes between different time points. The connectivity status between different nodes may not be well captured by this method.

Structural networks and functional networks are two interconnected concepts in the brain. Structural networks refer to the physical architecture formed by the connections between different brain regions via neural fibers, while functional networks describe the coordinated activity patterns of these regions during specific cognitive tasks [39]. Recent research has demonstrated that the relationship between structural and functional networks is not only static but also dynamic, and their interaction may have a significant impact on cognitive functions, behavioral performance, and disease states. In this study, we examined the cross‐scale connections of structure and function in SSNHL patients using SCN, multilayer network analysis, and DCM. Although SCN analysis revealed a significant group difference (FDR) in node efficiency for L.PreCG, the node efficiency of L.SFG (SSNHL = 0.2219, HC = 0.1884), L.SMA (SSNHL = 0.1903, HC = 0.1739), L.SPG (SSNHL = 0.1641, HC = 0.1635), and R.IPL (SSNHL = 0.1876, HC = 0.1636) was higher in SSNHL patients than in healthy controls, which aligns with the results from multilayer network analysis. This finding further supports the idea that structural networks provide the anatomical basis for the formation of functional networks. The neural connectivity patterns and network structures determine the pathways and rate of information flow, with functional networks facilitating communication through these connections. The integrity and efficiency of structural networks are often critical for the effective performance of brain functional networks. Additionally, while SSNHL did not show significant structural connectivity changes in L.SFG and L.SMA, the effective connectivity between L.SFG and L.SMA was significantly reduced. This suggests that disruptions in both cortical structure and function can contribute to cognitive impairments. The structural network serves as the physical foundation for functional networks and influences their organization and dynamics through its stability and plasticity [69]. Functional networks, in turn, may adapt to task demands or compensate for deficiencies in structural networks by adjusting the coordination patterns between brain regions. The interaction between these two networks is a fundamental mechanism enabling the brain's flexible response to environmental changes and task challenges [70].

Hearing loss leads to structural and functional remodeling of the cerebral cortex through two mechanisms: first, by consuming limited cognitive resources to compensate for the absence of sensory signals [4], and second, by the additional recruitment of higher‐order cognitive areas to mitigate the impact of hearing loss [71]. Both mechanisms are evident in our study results. Despite the short duration of SSNHL, leading to limited structural connectivity changes, this also poses a risk for some patients developing into long‐term hearing loss due to the inability to cure. Even in the early stages of hearing loss, the organism compensates for the effects of hearing loss through functional remodeling within the cerebral cortex. However, inevitably, this functional remodeling disrupts normal cognitive functions and causes inconvenience to patients in daily life. Thus, it is evident that while significant structural changes in the brain cortex have not occurred in the early stages of the disease, early intervention can delay or even prevent irreversible structural changes in the brain. Moreover, in the early stages of the disease, intervening in the areas of the brain cortex that exhibit functional changes may help treat or slow down the progression of the disease. A substantial body of evidence has demonstrated that transcranial magnetic stimulation (TMS) can improve tinnitus symptoms, and some studies have shown that repetitive TMS can enhance hearing function and tinnitus perception in patients with sudden sensorineural hearing loss. In future clinical work, this may facilitate the development of new treatment methods, such as neurostimulation techniques targeting brain network changes, as well as personalized treatment plans aimed at addressing both hearing and related cognitive or emotional issues.

This study has several limitations. First, the sample size of the study is small. The small sample size limits the generalizability and extrapolation of the study results, necessitating larger‐scale studies in the future to enhance the accuracy of the experimental outcomes. Secondly, this is a cross‐sectional study. Whether the structural and functional changes in SSNHL patients will reverse or worsen with the progression of the disease requires further confirmation in future research.

## 5 Conclusion

This study used SCN, multilayer brain network analysis, and DCM to delve into both structural and functional connectivity, exploring the cerebral cortex's structural and functional remodeling in patients with SSNHL. Results indicated that SSNHL patients had enhanced node efficiency in the L.PreCG. Additionally, an increased rate of nodal switching was observed at the levels of the L.SFG, L.SMA, L.SPG, R.IPL, R.SPG, and L.THA. Furthermore, the effective connectivity between the SPG and SMA was found to be reduced. The reallocation of cognitive resources due to hearing loss provides new insights into the potential mechanisms linking SSNHL with cognitive‐emotional disorders.

## Author Contributions

**Biao Li:** conceptualization, methodology, writing – review and editing, data curation. **Xiao‐Min Xu:** conceptualization, methodology, writing – review and editing, data curation. **Yuan‐Qing Wu:** methodology, software, data curation. **Yuan Feng:** investigation, data curation, writing – review and editing. **Yu‐Chen Chen:** writing – review and editing. **Richard Salvi:** writing – review and editing. **Jin‐Jing Xu:** conceptualization, methodology, writing – review and editing, data curation. **Jian‐Wei Qi:** writing – review and editing, data curation.

## Ethics Statement

The work described has been carried out in accordance with The Code of Ethics of the World Medical Association (Declaration of Helsinki) for experiments involving humans.

## Conflicts of Interest

The authors declare no conflicts of interest.

## Data Availability

The raw data generated in this study are available from the corresponding author on reasonable request and with the permission of the committee of Nanjing First Hospital.
